# Biochemical and Functional Insights into the Integrated Regulation of Innate Immune Cell Responses by Teleost Leukocyte Immune-Type Receptors

**DOI:** 10.3390/biology5010013

**Published:** 2016-03-08

**Authors:** Chenjie Fei, Joshua G. Pemberton, Dustin M. E. Lillico, Myron A. Zwozdesky, James L. Stafford

**Affiliations:** Department of Biological Sciences, University of Alberta, Edmonton, Alberta, T6G 2E9, Canada; chenjei@ualberta.ca (C.F.); jp28@ualberta.ca (J.G.P.); dlillico@ualberta.ca (D.M.E.L.); zwozdesky@ualberta.ca (M.A.Z.)

**Keywords:** teleost, immunoregulatory receptors, channel catfish, leukocyte immune-type receptors, innate immunity, phagocytosis, intracellular signaling, functional plasticity, tyrosine-based signaling motifs

## Abstract

Across vertebrates, innate immunity consists of a complex assortment of highly specialized cells capable of unleashing potent effector responses designed to destroy or mitigate foreign pathogens. The execution of various innate cellular behaviors such as phagocytosis, degranulation, or cell-mediated cytotoxicity are functionally indistinguishable when being performed by immune cells isolated from humans or teleost fishes; vertebrates that diverged from one another more than 450 million years ago. This suggests that vital components of the vertebrate innate defense machinery are conserved and investigating such processes in a range of model systems provides an important opportunity to identify fundamental features of vertebrate immunity. One characteristic that is highly conserved across vertebrate systems is that cellular immune responses are dependent on specialized immunoregulatory receptors that sense environmental stimuli and initiate intracellular cascades that can elicit appropriate effector responses. A wide variety of immunoregulatory receptor families have been extensively studied in mammals, and many have been identified as cell- and function-specific regulators of a range of innate responses. Although much less is known in fish, the growing database of genomic information has recently allowed for the identification of several immunoregulatory receptor gene families in teleosts. Many of these putative immunoregulatory receptors have yet to be assigned any specific role(s), and much of what is known has been based solely on structural and/or phylogenetic relationships with mammalian receptor families. As an attempt to address some of these shortcomings, this review will focus on our growing understanding of the functional roles played by specific members of the channel catfish (*Ictalurus punctatus*) leukocyte immune-type receptors (IpLITRs), which appear to be important regulators of several innate cellular responses via classical as well as unique biochemical signaling networks.

## 1. Introduction

Innate immune cells use a variety of receptor families to dynamically control the initiation, amplification, as well as termination of effector responses [1,2]. In general, immunoregulatory receptors are expressed at the cell surface and communicate extracellular stimuli to sophisticated, yet highly conserved, intracellular signaling networks that control potent antimicrobial functions [1,2]. The extracellular domains of each immunoregulatory receptor-type provide the interface for target recognition [3], whereas the transmembrane (TM) segments and cytoplasmic tail (CYT) regions translate binding events into a range of prototypical immune cell responses; characteristically, through a series of tyrosine-based signaling events [4]. Receptor-mediated signal transduction responses are vital for host protection from pathogens and, depending on the cell-type(s) activated and specific receptor(s) involved, include: degranulation, phagocytosis, cell-mediated cytotoxicity, and the production of bioactive molecules such as cytokines or various antimicrobial species.

Although it is well established that various teleost immune cell-types can execute the same range of effector responses that are performed by functional counterparts found in mammals, the specific immunoregulatory receptors and their associated signaling networks that control innate cellular defenses in fish remain unknown. For example, teleost immune cells can perform phagocytosis (macrophages and B cells) [5,6,7,8,9], cell-mediated cytotoxicity (Natural killer (NK)-like cells) [10,11,12], antigen presentation (macrophages) [13,14], and degranulation (mast cells and neutrophils) [15,16,17]. However, the immunoregulatory receptor systems controlling each of these important innate defenses have yet to be characterized in detail. In the past, these shortcomings were largely due to a lack of identified candidate genes, but the recent cloning and molecular characterization of an assortment of immune-related genes has significantly advanced our understanding of the immunoregulatory receptor families that exist in teleost fishes [18,19,20].

Many of the identified teleost receptors belong to the immunoglobulin superfamily (IgSF) and some of these share distant phylogenetic relationships and basic structural features with several mammalian immunoregulatory receptor families, including; Fc receptors (FcRs), FcR-like proteins (FcRLs), killer Ig-like receptors (KIRs), and leukocyte Ig-like receptors (LILRs). However, to date the orthologous relationships between the identified teleost genes and the known mammalian receptor sub-types remain to be established. One major factor that precludes defining orthology is the lack of identified ligands for teleost immunoregulatory receptors. Moreover, the discovery and characterization of novel immune-type receptors (NITRs) suggests that teleost immunity may also be in part regulated by unique receptor-types that are exclusive to bony fishes [21]. To our knowledge, teleost NITRs are not related to any immunoregulatory receptor families in amphibians, birds, or mammals; but, they do participate in NK cell-mediated allorecognition, implying they are the functional equivalents of mammalian NK receptors (NKRs) [22,23]. Further information regarding teleost NITRs can be found in [24,25,26]. Alternatively, teleost leukocyte immune-type receptors (LITRs), which were originally identified in the channel catfish (*Ictalurus punctatus*)*,* do share distant phylogenetic relationships with several families of mammalian IgSF members including FcRs, FcRLs, and various NKRs encoded within the leukocyte receptor complex (LRC) [27]. Information regarding the discovery, phylogenetic analyses, predicted structures, expression patterns, and putative binding partners of LITRs are reported in [27,28]; which we have also recently reviewed [20]. In addition, Table 1 presents the results of updated database searches for IpLITR-like proteins in other vertebrates, which further reinforces that these teleost proteins are distantly related to human FcRLs, FcRs, LILRs, KIRs, sialic acid-binding Ig-type lectins (Siglecs), carcinoembryonic antigen-related cell adhesion molecules (CEACAMs) as well as several IgSF members in rodents, birds, and frogs. It appears likely that the Ig-like domains contained within IpLITRs maintain some basal signatures of an ancestral IgSF that has since diversified throughout vertebrate evolution to become receptors involved in a broad range of vital immunoregulatory functions including antibody-binding, self- and non-self recognition, adhesion, and pathogen detection. Determining which of these important immunoregulatory functions, if any, are controlled by members of the IpLITR family will require further investigations. However, the fact that human immunoregulatory receptor sequences such as FcR/FcRLs, LILRs, and KIRs retrieve IpLITRs as the top matching sequences from basic local alignment search tool (BLAST) analyses indicates that IpLITRs are related to these various mammalian immune proteins. To further broaden the search for human immunoregulatory receptor-like sequences in additional fish species, selected protein sequences from Table 1 were also used in BLASTp searches against the teleost databases and representative matches from these searches are listed in Table 2. Although some of the human immunoregulatory receptor queries we used for searching the catfish databases did not retrieve any significant matches (*i.e.*, FcγRIIA, FcγRIIB, PIGR, KIR2DL1, Siglec-4, *etc*.; see Table 1) they did identify matches in several other fishes (Table 2). However, it remains to be determined whether or not these teleost sequences identified by BLASTp are indeed the functional equivalents of the human immunoregulatory receptor queries. Furthermore, since fish have both teleost-specific receptor families (*i.e.*, NITRs) and those that are distantly related to various mammalian immunoregulatory receptors belonging to the IgSF (*i.e.*, LITRs), it remains a formidable challenge to determine the precise roles of all of these receptor-types in teleost immunity.

Over the past decade, research in our lab has focused on characterizing the biochemistry and functionality of *Ictalurus punctatus* (Ip)LITRs with an emphasis on understanding how putative stimulatory and inhibitory receptor sub-types engage intracellular signaling responses to regulate immune cell actions. These efforts have revealed some expected, as well as unexpected details regarding IpLITR-mediated signaling events and their concomitant regulation of cellular immunity. By designing constructs that allow for the expression of IpLITRs in mammalian cells, we have taken advantage of epitope-tagging technology and commercially-available antibodies (Abs) to easily trigger these receptors in the absence of natural ligands and without developing IpLITR-specific monoclonal (m)Abs. Receptor chimeras that link the CYT regions of IpLITRs with the extracellular domains of mammalian receptors (*i.e.*, KIRs) have also allowed us to promote IpLITR-based signaling events using ligand-expressing target cells. Both of these strategies have assisted in defining key aspects of the stimulatory and inhibitory actions of IpLITRs as well as defining their unexpected functional versatility. It is important to qualify that our results do rely on heterologous expression of fish immunoregulatory proteins in surrogate, but well-characterized, mammalian cell systems. The interpretations made from the data collected from these types of experiments should always be considerate of this fact. Although this experimental strategy is not adequate for informing the actual *in vivo* activities of IpLITRs, our work represents the most detailed biochemical and functional examination of teleost immunoregulatory receptors to date. It is clear from our studies that IpLITRs are likely potent effectors of immune cell signaling and that they have the potential to regulate various innate cellular responses including NK cell-mediated killing, phagocytosis, degranulation, and cytokine secretion. Here we will review, in detail, the characterization of putative stimulatory and inhibitory IpLITR sub-types and provide frameworks for understanding the concept of IpLITR functional plasticity; a concept that has also emerged for several mammalian immunregulatory receptor-types.

## 2. Examination of Stimulatory IpLITR-Mediated Responses

### 2.1. Stimulatory Immunoregulatory Receptors

Stimulatory immunoregulatory receptors typically have short CYT regions and positively charged TM segments that facilitate intramolecular association with immunoreceptor tyrosine-based activation motif (ITAM)-containing adaptors [29]. For example, the Fc receptor (FcR) common gamma chain (FcRγ) associates with members of the FcR family and is requisite for their surface expression and signaling capacity [30]. Engagement of FcRs by binding the Fc region of immunoglobulin induces the activation of Src family kinases (SFKs) that phosphorylate tyrosine residues within the ITAMs of the associated FcRγ [30]. Phosphorylation of the ITAM is followed by the rapid recruitment and activation of spleen tyrosine kinase (Syk) [31,32], which then triggers several canonical transduction cascades leading to the induction of effector responses such as phagocytosis, degranulation, and cytokine secretion [33,34,35,36,37,38]. Alternatively, the adaptor protein DAP12 plays a key role in cytotoxicity mediated by natural killer (NK) cells via its association with activating KIRs [39] and other NKRs [40]. As with FcRγ, formation of KIR-DAP12 complexes is dependent on electrostatic interactions facilitated by the oppositely charged TM segments of the receptor (positively charged) and adaptor (negatively charged) proteins [29]. A full complement of ITAM-containing adaptor molecules have been identified in fish, which includes homologues of FcRγ and DAP12 [41]. This suggests that the mode of receptor-mediated immune cell activation in teleosts is likely similar to what occurs in mammals.

### 2.2. Stimulatory IpLITRs Demonstrate a Unique Ability to Associate with Intracellular Signaling Adaptors

The characteristic TM segment of IpLITR 2.6b, a representative stimulatory IpLITR-type, contains a positively charged lysine residue and a short CYT region that lacks canonical tyrosine-based signaling motifs [27]. Therefore, we predicted that this receptor required an adaptor protein to facilitate its cell surface expression and to stimulate kinase-dependent intracellular signaling networks. We tested this directly using HEK 293T cells co-transfected with hemagglutinin (HA)-tagged IpLITR 2.6b, and demonstrated that this receptor co-immunoprecipitated with the ITAM-containing IpFcRγ, IpFcRγ-L, and IpCD3ζ-L signaling adaptors but not with IpDAP12 [42]. In mammals, stimulatory receptor-types that have lysine residues in their TM segments bind DAP12 but not FcRγ [29]. A significant increase in the cell surface expression of IpLITR 2.6b in the presence of IpFcRγ or IpFcRγ-L, but not IpCD3ζ-L, was observed; indicating that association with an appropriate adaptor is required for localization of the active signaling complex within the plasma membrane [42,43]. These studies also demonstrated that IpLITR 2.6b associated with heterodimeric complexes composed of IpFcRγ-L and IpCD3ζ-L (but not IpDAP12) [42], which has been previously observed for FcRγ-CD3ζ heterodimers during the control of FcR-dependent signal transduction in mammals [44]. Mutation of the lysine residue within the TM segment of IpLITR 2.6b to arginine or an uncharged alanine residue had no effect on either surface expression or adaptor recruitment. In addition, the neutralized TM segment of IpLITR 2.6b not only maintained its ability to bind IpFcRγ-L and IpCD3ζ-L, but also acquired the ability to bind IpDAP12, which significantly augmented the cell surface expression of IpLITR 2.6b [42]. These observations suggest that, contrary to what has been reported for stimulatory immunoregulatory receptor-adaptor associations in mammals [29], a charged TM segment is not a prerequisite for adaptor recruitment to IpLITR 2.6b. However, the identity of the residues within the TM segment does appear to define the specificity of IpLITR-adaptor protein interactions. Alternatively, neutralizing the negatively charged TM region of the signaling adaptor IpFcRγ-L completely abrogated assembly with IpLITR 2.6b and inhibited the surface expression of the IpLITR 2.6b-IpFcRγ-L complex [42]. These data strongly suggested that the negatively charged TM segment of the channel catfish adaptor molecule was an important requirement for IpLITR-adaptor assembly. Overall, these biochemical studies confirmed that a putative stimulatory IpLITR-type associated with teleost-specific ITAM-containing adaptors and provided interesting new details regarding the specificity of these interactions. Our next objective was to examine the functional implications of IpLITR 2.6b-IpFcRγ-L interactions by testing the ability of this immunoregulatory protein complex to stimulate various innate immune cell activities.

### 2.3. Induction of Intracellular Signaling and Immune Cell Activation by a Stimulatory IpLITR-Adaptor Protein Complex

To directly examine the functional activities of the IpLITR 2.6b-IpFcRγ-L signaling subunit we generated an N-terminal HA-tagged chimeric receptor construct consisting of the extracellular region of IpLITR 2.6b fused with the TM segment and ITAM-containing CYT region of IpFcRγ-L [45]. The IpLITR 2.6b/IpFcRγ-L chimera was then transfected and stably-expressed in the rat basophilic leukemia (RBL-2H3) cell line, where it could then be triggered using anti-HA mAbs. Using this approach, we demonstrated that IpLITR 2.6b/IpFcRγ-L activated intracellular signaling mediators and that it was a potent stimulator of the RBL-2H3 degranulation response [45]. Mutating the IpLITR 2.6b/IpFcRγ-L ITAM tyrosine residues to phenylalanine completely blocked functional outputs, reinforcing the ITAM-dependent nature of these cellular responses. Using phospho-specific Abs and Western blotting, we also observed a unique time-course of extracellular signal-regulated kinase (ERK) 1/2 and protein kinase B (Akt) phosphorylation after IpLITR 2.6b/IpFcRγ-L triggering, which did not occur using the ITAM mutated receptor [45]. Furthermore, pharmacological inhibitors of SFKs, phosphatidylinositol 3-kinases (PI3Ks), mitogen activated protein kinase (MAPK) kinase (MEK1 and MEK2), and protein kinase Cs (PKC) significantly inhibited IpLITR 2.6b/IpFcRγ-L-induced degranulation [45]. In comparison, selective inhibitors of the c-Jun N-terminal kinases (JNKs) and p38 MAPK pathways had no effect on stimulated IpLITR 2.6b/IpFcRγ-L cellular responses. Overall, these studies provided the first functional information for a putative stimulatory IpLITR-adaptor complex and suggested that the ITAM-dependent signaling activated by IpLITR 2.6b/IpFcRγ-L occurs through classical kinase-dependent intracellular cascades [45]. A proposed ITAM-dependent signaling mechanism utilized by IpLITR 2.6b was summarized previously in [45].

The functional capacity of IpLITR 2.6b/IpFcRγ-L was further examined using a MAPK signaling array as well as cytokine secretion profiling. Anti-HA crosslinking of IpLITR 2.6b/IpFcRγ-L increased phosphorylation of several intracellular signaling molecules, including ERK1/2, GSK-3α/β, GSK-3β, RSK1, CREB, JNK (pan), MEK6, MSK2, p38δ, and Akt2 [46]. This provided a broader view of the intracellular signaling components phosphorylated downstream of IpLITR 2.6b/IpFcRγ-L triggering. These studies also demonstrated that cells activated via IpLITR 2.6b/IpFcRγ-L secreted interleukin (IL)-3, IL-4, IL-6, and tumor necrosis factor-α (TNF-α) at levels comparable to what we observed following activation of the cells with phorbol myristate acetate, calcium (Ca^2+^) ionophore, or stimulation via triggering the endogenous FcεRI [46]. This suggested that IpLITR 2.6b/IpFcRγ-L-mediated signaling events coordinately induce both cellular degranulation and cytokine secretion over a comparable temporal scale. Moreover, when the IpLITR 2.6b/IpFcRγ-L chimeric construct was triggered with anti-HA mAb opsonized beads, we observed a potent phagocytic response that was dependent on extracellular Ca^2+^ availability and actin polymerization [46]; thus adding another functional dimension to IpLITR 2.6b/IpFcRγ-L activation.

In mammals, phagocytic receptors such as FcRs also associate with the ITAM-containing adaptor FcRγ-chain [34,47,48]. Phosphorylation of FcRγ-chain ITAMs by SFKs leading to the recruitment of Syk, which then serves as the main effector of FcR-mediated phagocytic signaling by directly binding to and/or phosphorylating specific downstream intracellular components [49,50]. Classically, the mediators recruited to FcR-FcRγ complexes include isoforms of PI3Ks, Vav, and Rho family GTPases [51,52,53,54]; in particular, Rac1 and Cdc42 dynamically control actin polymerization and are required for phagocytic cup formation as well as pseudopod extension [55,56]. Selective pharmacological inhibitors of these mediators were profiled in phagocytic assays in order to obtain detailed information regarding the biochemical pathways that facilitate IpLITR 2.6b/IpFcRγ-mediated phagocytosis and to compare these mechanisms with the classical mammalian FcR phagocytic mode [57]. Phagocytic activity was measured using a flow cytometric-based assay and 4.5 µm fluorescent polystyrene microsphere targets. IpLITR 2.6b/IpFcRγ-L-mediated phagocytosis relied on a similar subset of intracellular effectors for target engulfment and was comparable to the ITAM- and SFK-dependent mode of phagocytosis utilized by mammalian FcRs [49,50,51]. Specifically, the IpLITR 2.6b/IpFcRγ-L phagocytic response was inhibited using small molecule inhibitors that targeted SFKs, Syk, PI3Ks, Cdc42, Rac1/2/3, phosphoinositide-dependent kinase 1 (PDK1), Akt, PKC, MEK1/2, and F-actin polymerization [57]. These data provide further biochemical details describing the phagocytic pathway engaged by a teleost immunoregulatory receptor. The marked similarities observed between IpLITR- and mammalian FcR-mediated phagocytosis indicates that ITAM-dependent stimulatory signaling in vertebrates may represent a common, and perhaps evolutionarily conserved, signaling mode for active target engulfment.

## 3. Examination of Inhibitory IpLITR-Mediated Responses

### 3.1. Inhibitory Immunoregulatory Receptors

Inhibitory immunoregulatory receptors establish the activation threshold of immune cells and attenuate stimulatory immunoregulatory receptor-induced effector functions. Inhibitory actions are primarily dependent on immunoreceptor tyrosine-based inhibition motifs (ITIMs) within the CYT regions of putative inhibitory receptors [58,59,60]. Following receptor triggering, phosphorylation of the tyrosine residue embedded within ITIMs (S/I/V/LxYxxI/V/L) promotes binding of SH2 domain-containing cytoplasmic phosphatases (SHP-1, SHP-2, and SH2-domain containing inositol 5-phosphatase 1 or 2; SHIP1/2). Activated phosphatases dephosphorylate important proximal signaling proteins and transduction molecules in order to block the transmission of kinase-mediated signaling events and inhibit immune cell effector functions such as phagocytosis, degranulation, and NK cell-mediated killing responses [60,61,62]. Some inhibitory receptors also contain immunoreceptor tyrosine-based switch motifs (ITSMs; [63]) in the CYT region that can similarly inhibit cellular activation via phosphatase recruitment, but ITSMs can also alter cellular signaling via phosphatase-independent mechanisms following the selective recruitment of adaptor proteins such as SH2 domain protein 1A (SH2D1A) and Ewing’s sarcoma (EWS)-activated transcript 2 (EAT-2) [63]. Not surprisingly, many of the identified IpLITRs contain ITIMs and/or ITSMs, and we have investigated the biochemical and functional properties of putative inhibitory IpLITRs, which will be reviewed in this section.

### 3.2. Inhibitory IpLITRs Contain ITIMs and Recruit Protein Tyrosine Phosphatases

We first examined if ITIM-containing IpLITR-types recruited SHP-1 and/or SHP-2 after receptor engagement and phosphorylation of their CYT tyrosines. To do this, expression constructs were generated by fusing the extracellular domain and TM segments of the human NK cell receptor KIR2DL3 to the tyrosine-containing CYT regions of two selected putative inhibitory IpLITR-types, IpLITR 1.2a and IpLITR 1.1b [64]. The extracellular region of KIR2DL3 was used in the fusion constructs since the biological ligand for this receptor is known and readily available (*i.e.*, HLA-Cw3) [60]. The KIR_ED_-LITR_CYT_ constructs were originally examined using transient expression experiments in HEK 293T cells [64]. Here we demonstrated that following tyrosine phosphorylation, the CYT regions of IpLITR 1.2a and IpLITR 1.1b recruited SHP-1 and SHP-2 in an ITIM-dependent manner. IpLITR 1.1b also had a unique tyrosine-containing TM proximal region lacking classic inhibitory or stimulatory tyrosine-based signaling motifs that, in our studies, did not bind SHP-1 or SHP-2 [64]. After transfection and stable expression of these KIR_ED_-LITR_CYT_ constructs in mammalian NK-like cells, we anticipated that HLA-Cw3-positive target cells would effectively engage the chimeric proteins; allowing us to directly examine IpLITR-mediated inhibitory signaling. This is important since, as outlined above, the natural ligands for IpLITRs are unknown. Using this novel system, we demonstrated that IpLITR-mediated signaling events could influence cytotoxic responses and, as predicted, that IpLITR engagement could abrogate NK cell-mediated killing responses [65]. Unexpectedly, we also demonstrated that this inhibitory signaling occurred via both SHP-dependent and -independent mechanisms.

### 3.3. Inhibitory IpLITRs Abrogate the NK Cell Killing Response via Both SHP-1-Dependent and -Independent Signaling Pathways

The inhibitory functions of IpLITRs were examined using a vaccinia virus system to express KIR_ED_-LITR 1.2a_CYT_ and KIR_ED_-LITR 1.1b_CYT_ in mouse spleen-derived cytotoxic lymphocytes [65]. Unlike our previous studies, this allowed us to determine the specific effects of IpLITR-induced signaling on lymphocyte cytotoxicity using B cell targets expressing HLA-Cw3 (e.g.*,* 721.221 cells). The main objectives of this study were to examine the inhibitory signaling pathways activated by IpLITR 1.2a and IpLITR 1.1b as well as to determine if the unique proximal region of the IpLITR 1.1b CYT differentially contributed to the regulation of NK cell-mediated cytotoxicity. We observed that the CYT regions of IpLITR 1.2a and IpLITR 1.1b both contributed to the inhibition of lymphocyte-mediated target cell killing and determined that for IpLITR 1.2a, this was a SHP-dependent mechanism [65]. However, the inhibitory function mediated by the CYT region of IpLITR 1.1b was not affected by co-expression with a catalytically inactive SHP-1 recombinant protein, suggesting that although IpLITR 1.1b binds SHP-1, its inhibitory activity is not dependent on the catalytic activity of this phosphatase [65].

The IpLITR 1.1b CYT region contains six tyrosine residues, evenly distributed between its membrane proximal (Y^433^, Y^453^, and Y^463^) and distal (Y^477^, Y^499^, and Y^503^) regions [64]. The distal region contains two ITIMs located at Y^477^ and Y^499^ and one overlapping ITSM (Y^503^) and when tested as a separate construct, IpLITR 1.1b CYT_DISTAL_ displayed potent inhibitory activity that was dependent on SHP-1 [65]. We also generated a construct encoding only the CYT proximal region of IpLITR 1.1b and, surprisingly, this receptor variant also blocked NK cell-mediated killing responses despite the fact this construct did not contain any ITIMs or ITSMs and could not recruit SHP-1 or SHP-2 [64,65]. This revealed that the SHP-independent inhibitory pathway being activated by IpLITR 1.1b was likely facilitated by one of the tyrosine residues present within its unique proximal CYT region. We then determined that the peptide sequence AV**Y**^453^AQV in the IpLITR 1.1b CYT_PROXIMAL_ closely matched the consensus-binding motif **Y** [T/**A**/S] [K/R/**Q**/N] [M/I/**V**/R], which is required for recruiting the C-terminal Src kinase (Csk) [66].

In mammals, Csk has been identified as a major endogenous inhibitor of SFK-mediated cellular activities [67,68] and, in general, is an important regulator of intracellular signaling events. However, the ability of Csk to negatively influence cellular immune responses has only recently been recognized [69,70]. The inhibitory actions of Csk are dependent on its ability to phosphorylate a conserved tyrosine residue located at the C-terminus of SFKs (*i.e.*, Lyn, Fyn, Hck, *etc*.), which inhibits SFK catalytic activities [67,68]. Kinase-mediated inhibitory signaling opposes the classical phosphatase-driven attenuation of immune cell responses, but is likely equally important in terms of the overall regulation of immunity. Using co-immunoprecipitation and site-directed mutagenesis, we confirmed that Y^453^ was indeed required for Csk binding to the IpLITR 1.1b proximal CYT region as well as the inhibitory activity of IpLITR 1.1b [65]. Recruitment of Csk to the proximal CYT region of IpLITR 1.1b would appropriately localize this kinase for targeted phosphorylation of SFKs rendering them inactive and thus abrogating the early signaling events required for cellular activation, such as NK cell-mediated killing. These results were the first functional studies reported for an inhibitory ITIM-containing IpLITR and the first to suggest the involvement of Csk as a novel inhibitory signaling mechanism used by a teleost immunoregulatory receptor [65]. Furthermore, the unique proximal and distal inhibitory signaling mechanisms mediated by the IpLITR 1.1b CYT reveal marked versatility in the signaling properties within a single LITR. In the subsequent sections, we will review aspects of IpLITR functional plasticity in more detail.

## 4. Examination of IpLITR-Mediated Functional Plasticity

### 4.1. Functional Plasticity and Immunoregulatory Receptors

The classification of immunoregulatory receptors as inhibitory or stimulatory has depended primarily on whether or not they contain key signaling motifs within the CYT region such as ITIMs (inhibitory receptors) or ITAMs (stimulatory receptors). However, the functional outcome of immunoregulatory receptor engagement does not always coincide with the presence of these canonical motifs, as alternative mechanisms of ITIM- or ITAM-dependent signaling events may contribute to functional versatility. For example, there are several reports demonstrating that ITIM-encoding receptors can also stimulate immune cell responses through a wide variety of functionally distinct signaling mechanisms [71,72,73,74,75,76]. In addition, inhibitory actions triggered by phosphorylated ITAMs have also been described [77,78,79,80,81]. Functional plasticity in the transduction events controlled by modular protein domains likely serves as an important regulatory mechanism for the fine control of innate immune cell effector functions. Integrated receptor-mediated regulation of signal transduction is also likely to be dependent on many additional factors including the immune cell-type(s) involved as well as the available repertoire of intracellular adaptor and effector molecules. The magnitude and duration of immunoregulatory receptor activation by natural ligands may also play a key role in the type of functional outcomes that occur following receptor engagement. We have already demonstrated that IpLITR 1.1b inhibits NK cell-mediated killing using a classical ITIM- and SHP-dependent mechanism that was regulated by its distal CYT region [65]. Interestingly, this receptor also abrogated NK cell responses using an ITIM-independent mechanism that relied on the recruitment of Csk to a tyrosine residue located in the proximal CYT region [65]. Although the signaling versatility observed contributed to the same functional outcome, it occurred through two distinct intracellular mechanisms. Moreover, we recently demonstrated that IpLITR 1.1b could potently activate a phagocytic response in RBL-2H3 cells (a representative myeloid cell type), suggesting that the differential recruitment of signaling mediators in specific immune cell types (*i.e.*, lymphoid *vs.* myeloid) may facilitate a context-dependent plasticity during the receptor-mediated control of cellular processes [46]. Our findings using IpLITRs agree with observations from mammalian models describing alternative mechanisms of immunoregulatory receptor-mediated signaling that extend beyond their putative classifications as strictly inhibitory or stimulatory receptor-types. Overall, alternative stimulatory signaling downstream of IpLITR 1.1b activation provides the basis for the examination of IpLITR functional plasticity as an important immunomodulatory mechanism controlled by these teleost receptors.

### 4.2. Induction of Phagocytosis and Stimulatory Signaling by an Inhibitory IpLITR

In the original description of IpLITR 1.1b functional plasticity, we demonstrated that this receptor stimulated phagocytosis and induced phosphorylation of important signal transduction targets, including ERK1/2 and Akt [46]. However, unlike IpLITR 2.6b/IpFcRγ-L, IpLITR 1.1b did not induce the secretion of cytokines and the kinetics of IpLITR 1.1b-induced phosphorylation of ERK1/2 and Akt was significantly different from the ITAM-mediated responses. IpLITR 1.1b stimulatory activities were also independent of any association with an ITAM-containing adaptor protein, and phagocytic activity stimulated by IpLITR 1.1b was insensitive to treatment with the extracellular Ca^2+^ chelator EDTA, which further distinguished IpLITR 1.1b-mediated signaling from the IpLITR 2.6b/IpFcRγ-L pathway [46]. Finally, without the CYT region, IpLITR 1.1b was still expressed on the cell surface but it no longer stimulated a phagocytic response; indicating that the signal to initiate phagocytosis was due to events promoted by residues contained within the CYT [46]. To further understand the requirements for IpLITR 1.1b-mediated phagocytosis, we again considered the unique proximal and distal composition of tyrosine residues within the CYT in an attempt to derive a mechanism that could be examined in detail using biochemical and functional assays. The first step in this process was to use bioinformatics to predict what intracellular molecules might bind to the IpLITR 1.1b CYT and to hypothesize how these transduction elements would participate in the activation of machinery required for actin polymerization as well as plasma membrane remodeling during target engulfment. Since IpLITR 1.1b does not contain a classical ITAM, or any other recognizable stimulatory motifs that are known to facilitate phagocytosis [64], an alternative mode for the intracellular stimulation of IpLITR 1.1b-mediated phagocytosis was considered. Based on the results described in [46], this mechanism will be explained further below.

**Figure 1:**
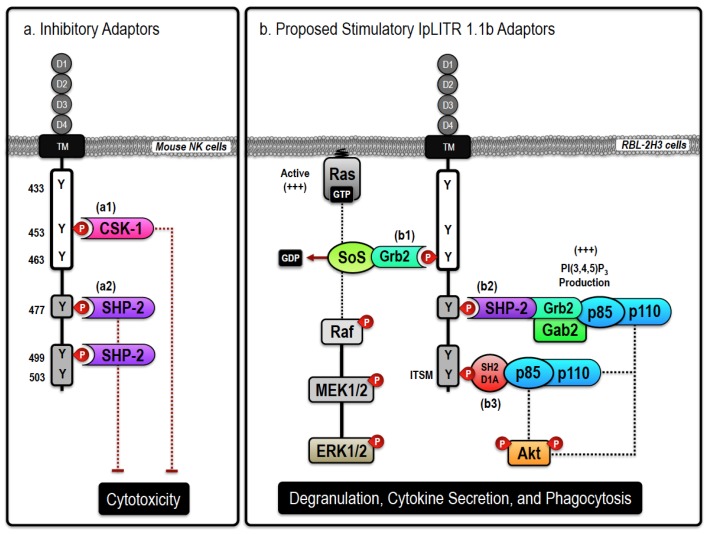
Proposed inhibitory and stimulatory (*Ictalurus punctatus*) leukocyte immune-type receptors (IpLITR) 1.1b-mediated intracellular signaling events. Schematic representation of the proposed inhibitory (**a**) and stimulatory (**b**) signaling events mediated by IpLITR 1.1b in transfected cells. The cytoplasmic tail (CYT) region of IpLITR 1.1b contains six tyrosine residues (Y^433^, Y^453^, Y^463^, Y^477^, Y^499^, and Y^503^) that, when phosphorylated, serve as potential docking sites for various intracellular signaling adaptors. (**a**) In mouse natural killer (NK) cells, we demonstrated that engagement of IpLITR 1.1b caused a potent inhibition of NK cell-mediated cytotoxicity due to the recruitment of Csk-1 at Y^453^ (**a1**) or the binding of SH2 domain-containing cytoplasmic phosphatases (SHP) at Y^477^ and/or Y^499^, which are in immunoreceptor tyrosine-based inhibition motifs (ITIMs) (**a2**) [64,65]. The immunoreceptor tyrosine-based switch motifs (ITSM) located at Y^503^ may also recruit SHP phosphatases but this has not been examined. (**b**) IpLITR 1.1b engagement also induced phosphorylation of ERK1/2 and Akt as well as promoted phagocytosis in transfected rat basophilic leukemia (RBL)-2H3 cells [46]. These stimulatory effector cell functions could be mediated by the following mechanisms; (**b1**) direct recruitment of growth factor receptor-bound 2 (Grb2) to the YxN motif at Y^463^ may mediate the recruitment, and associated GEF activity, of SoS or the Gab2/class I PI3K (p85/p110) signaling complex. SoS is known to stimulate the accumulation of GTP-loaded Ras that would facilitate the stepwise phosphorylation of the Raf-MEK-ERK cassette. Alternatively, the Gab2 adaptor can localize class I PI3K activation to allow for targeted accumulation of the important signal transduction molecule phosphatidylinositol 3,4,5-trisphosphate (PI(3,4,5)P_3_); (**b2**) ITIM-mediated recruitment of SHP-2 at Y^477^ or Y^499^, could recruit Grb2 and Gab2 allowing for the association of holomeric class I PI3Ks (p85/p110) leading to Akt phosphorylation and induction of phagocytosis. SHP-2-dependent recruitment of class I PI3Ks could also occur at the C-terminal ITSM located at Y^503^. SHP-2-dependent Grb2 recruitment may also trigger the SoS/Ras/Raf/MEK-dependent activation of ERK1/2; (**b3**) SH2D1A-mediated binding of PI3K (p85/p110) to the ITSM at Y^503^ is also possible. In general, class I PI3K activation can result in Akt phosphorylation or the recruitment of other PI(3,4,5)P_3_-dependent signaling proteins, including Vav, that are known to regulate phagocytosis. In addition, ITSM-mediated signaling can also recruit the adaptor EAT-2, closely related to SH2D1A, which is not shown here. For clarity, the role of signaling events dependent upon extracellular Ca^2+^ entry or intracellular Ca^2+^ mobilization have also been excluded.

One potential mechanism derived from mammalian models hypothesizes that the ITSM-containing distal region of the CYT is primarily responsible for IpLITR 1.1b functional plasticity. For example, ITSMs can bind SHPs that commonly mediate phosphatase-dependent cellular inhibition [82]. However, as their name suggests, this motif can also alter conformation to selectively recruit stimulatory adaptor proteins including SH2D1A and EAT-2 that typically activate, rather than inhibit, effector functions in immune cells [83]. In particular, ITSMs can facilitate stimulatory signaling through SH2D1A-dependent recruitment and activation of the p85 subunit of class IA PI3Ks [84,85], which can then induce Akt phosphorylation downstream of phosphatidylinositol 3,4,5-trisphosphate (PI(3,4,5)P_3_) production as well as directly modulate phagocytosis [63]. ITIM- and ITSM-mediated induction of cellular activation has also been shown to involve SHP-2 [86], which instead of abrogating downstream signaling, can serve as a key scaffold for proteins involved in cellular responses. In this scenario, SHP-2 recruitment to ITIMs and/or ITSMs results in phosphorylation at a C-terminal tyrosine that then serves as a binding site for the SH2-containing adaptor molecule growth factor receptor-bound 2 (Grb2) [87,88]. Grb2 associates with members of the Dab/Dos family of scaffolding proteins, known as the Grb2-associated binders (Gabs). When phosphorylated, Gab2 has been shown to bind and activate class IA PI3Ks, again resulting in the recruitment and phosphorylation of downstream targets that include PDK1 and Akt as well as other components of the phagocytic machinery [89,90,91,92]. During IpLITR 1.1b-mediated phagocytosis, we predict that Grb2 recruitment to the activated receptor may rely on SHP-2 binding to the ITIMs (Y^477^ and/or Y^499^)_,_ and/or the ITSM (Y^503^). SHP-2 recruitment would allow for the association of larger protein heteromers, including potential interactions with Gab2 and formation of the SHP-2-Grb2-Gab2 ternary signaling complex that would allow for the subsequent activation of class I PI3Ks [88,93,94,95]. The involvement of SHP-2 as an intracellular scaffold capable of engaging PI(3,4,5)P_3_-dependent cellular activation pathways provides an explanation for context-dependent functional outcomes contrasting our previously reported SHP-dependent inhibition of NK cell killing induced by IpLITR 1.1b [65]. An overall summary schematic of this proposed signaling mechanism is provided in Figure 1.

The stimulatory actions of IpLITR 1.1b are unlikely limited to only the distal CYT region containing the ITIMs and ITSM. As also outlined in Figure 1, Y^463^ matches the known Grb2-binding motif (YxN) that would facilitate direct Grb2 binding to the CYT proximal region of IpLITR 1.1b [66]. As already mentioned, Grb2 is an important intracellular scaffold that is also known to associate with guanine nucleotide exchange factors (GEF), such as Son of Sevenless (SoS), which interacts with the SH3 domain of Grb2 via a polyproline-rich sequence [96]. Formation of the Grb2-SoS signaling complex also commonly involves targeting the GEF activity of SoS to the membrane-associated Ras superfamily of small GTPases, subsequently leading to the activation of the Raf-MEK-ERK signaling cassette [97,98]. Therefore, direct or indirect (via SHP-2) recruitment of Grb2 to the proximal and/or distal CYT regions of IpLITR 1.1b, respectively, may provide explanations for how this receptor can induce the downstream activation of both ERK1/2 and Akt signaling in RBL-2H3 cells. Alternatively, Grb2 binding directly to phosphorylated Y^463^ (*i.e.*, YxN motif) during phagocytosis could also recruit Gab2 and the class I PI3Ks. Gab2-dependent recruitment and activation of class IA PI3K heterodimers (p85-p110) would increase the local generation of PI(3,4,5)P_3_, which is required for actin-dependent extension of pseudopods and the engulfment of large extracellular particles [91,95,99,100]. IpLITR 1.1b-induced production of PI(3,4,5)P_3_ could also attract additional pleckstrin homology (PH) domain-containing adaptors like Vav, another important GEF, that directly bind to plasma membrane PI(3,4,5)P_3_ [101]. Vav activation stimulates Rho family GTPases including Cdc42, Rac1, and RhoA that control local actin polymerization during the formation of lamellipodia required for phagocytosis [54]. Control of Rho family GTPase activity by Vav has also been shown to play an important role in FcRγ-, complement receptor-, and CEACAM-mediated phagocytosis [101,102]. It still remains to be seen if Vav-dependent regulation of Rho family GTPases participates in IpLITR-mediated phagocytosis, however, interactions between IpLITR 1.1b and many of the molecules implicated above are the focus of ongoing investigations in our lab.

In addition to Csk, SHP-1, and SHP-2 reported in [65], we have biochemical evidence for the binding of Grb2, SH2D1A, and the class IA PI3K regulatory subunit p85 to the CYT region of IpLITR 1.1b (unpublished observations; Zwozdesky and Stafford). Overall, our studies using IpLITR 1.1b are the first to demonstrate functional plasticity for an ITIM-containing teleost immunoregulatory receptor. At present, the precise mechanisms of IpLITR 1.1b-induced phagocytosis are unknown, but the events leading to this outcome are clearly distinct from those facilitated by the prototypical ITAM-dependent pathway used by IpLITR 2.6b and may ultimately represent a novel cellular mode of actin-dependent target engulfment.

### 4.3. IpLITRs Activate Distinct Phagocytic Modes: Further Insights into IpLITR 1.1b-Mediated Functional Plasticity

**Figure 2:**
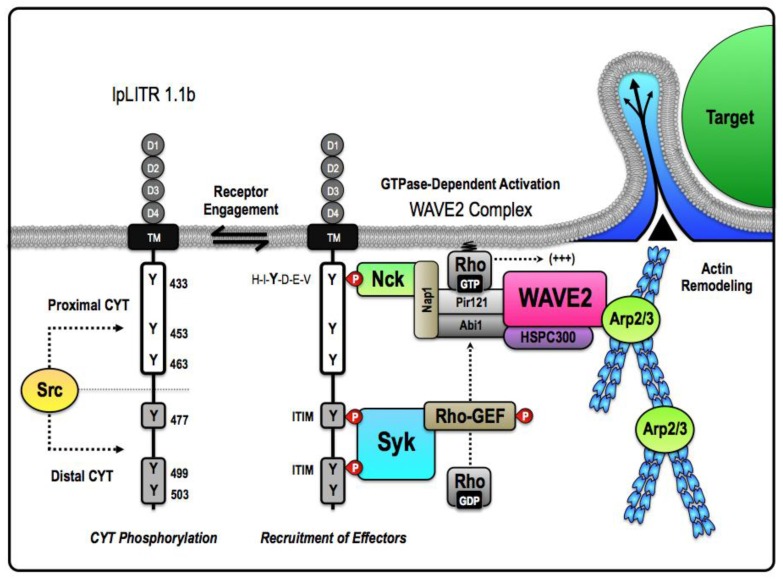
Proposed mechanism for an ITAM-independent target acquisition and engulfment pathway facilitated by pLITR 1.1b. The unique short-circuited version of phagocytosis exhibited by IpLITR 1.1b-expressing cells likely requires that the proximal and distal regions of the IpLITR 1.1b CYT differentially participate in the recruitment and activation of select phagocytic effectors. Our results suggest that IpLITR 1.1b-mediated regulation of the actin polymerization machinery is dependent upon the catalytic activity of the Src and spleen tyrosine kinase (Syk) families of intracellular kinases [57]. We hypothesize that Src serves to place IpLITR 1.1b in a primed state that facilitates basal or constitutive coupling of IpLITR 1.1b to the minimal intracellular machinery required for target acquisition and phagocytic cup extension. In this model, the cytosolic adaptor non-catalytic region of tyrosine kinase adaptor protein 1 (Nck) is recruited to the consensus interaction motif H-I-Y-D-E-V located at Y^433^ in the proximal CYT region of IpLITR 1.1b. Nck has been shown to associate with the WAVE2 complex; a highly conserved pentameric heterocomplex that dynamically regulates Arp2/3-dependent actin polymerization [104]. Importantly, in mammalian cells, WAVE2 is expressed ubiquitously and found as a complex with four other proteins: Pir121, Nap1, Abi-1, and HSPC300 [104]. The mature WAVE2 complex is basally inactive and directly interacts with the SH3 domain of Nck through Nap1 [103,105]. Activation of the WAVE2 complex requires state-specific phosphorylation as well as interactions with GTP-bound Rho superfamily proteins, most commonly Rac [106]. As a result, we propose that the assembly of the Nck-WAVE2 complex within the proximal CYT region of IpLITR 1.1b could be coupled to recruitment of a stimulatory Rho-GEF within the distal CYT region. In particular, the spacing of the tyrosines in IpLITR 1.1b suggest that Syk could be recruited to two tandem ITIM motifs at Y^477^ and Y^499^ in the distal CYT region. Based on comparisons with other phagocytic receptors, we suspect that activation of the cytosolic Rho-GEF could be Syk-dependent. Syk activation of the Rho-GEF would provide the necessary catalyst for rapid actin-driven membrane protrusions via the WAVE2 complex. Together, this mechanism would encompass the minimal machinery required for PI3K-independent target capture pathway by IpLITR 1.1b.

Details of an alternative ITAM-independent IpLITR-induced phagocytic pathway were revealed in [57]. These studies indicated that despite convergence on the control of actin polymerization dynamics, IpLITR 1.1b clearly operates independently of several of the key components of the ITAM-dependent signaling machinery. IpLITR 1.1b-expressing cells also displayed a unique target acquisition and engulfment phenotype that was not observed during IpLITR 2.6b/IpFcRγ-induced phagocytosis. Phagocyte phenotypes were examined by confocal microscopy, which demonstrated that IpLITR 1.1b-expressing cells often formed extended membranous protrusions that captured their targets in phagocytic cup-like structures but failed to completely internalize them. Incomplete target engulfment is indicative of a stalled phagocytic phenotype that was often observed during IpLITR 1.1b-mediated phagocytosis (~44% of cells examined), but was rarely observed for the IpLITR 2.6b/IpFcRγ-L-expressing cells (~3% of cells) that readily internalized extracellular targets (*i.e.*, ~60% of the cells analyzed had completely engulfed two or more beads) [57]. Although the capture and partial engulfment phenotype was common during IpLITR 1.1b-mediated phagocytosis, ~30% of the analyzed cells internalized at least one 4.5 µm bead. Surprisingly, while incubations at 22°C inhibited IpLITR 2.6b/IpFcRγ-L, this lower temperature had no effect on the ability of IpLITR 1.1b-expressing cells to capture and partially engulf beads; although the depressed temperature did reduce the ability of IpLITR 1.1b-expressing cells to completely engulf extracellular targets (*i.e.*, from~30% to 10% internalization). Moreover, while small molecule inhibitors of major kinase and GTPase signaling systems had significant inhibitory effects on the IpLITR 2.6b/IpFcRγ-L-mediated phagocytic pathway (described above), IpLITR 1.1b-induced phagocytosis was insensitive to the majority of these pharmacological inhibitors. For example, IpLITR 1.1b phagocytosis was only significantly blocked by treatment with inhibitors of SFKs, Syk, and F-actin nucleation, whereas inhibition of PDK1, Cdc42, Rac, PI3Ks, and PKCs were ineffective [57]. Based on these findings, we propose a distinctive cellular model of phagocytosis for IpLITR 1.1b that requires a minimal set of intracellular signaling components that directly associate with the actin polymerization machinery (Figure 2). We also hypothesize that the proximal and distal regions of the IpLITR 1.1b CYT may differentially participate in the recruitment and activation of phagocytic effectors. For IpLITR 1.1b to activate phagocytosis, our recent results [57] suggest that it must stimulate actin polymerization and may require the catalytic activity of the SFK and Syk families of intracellular kinases. Therefore, we propose that CYT proximal region recruits the actin polymerization machinery into the vicinity of a Syk-dependent activation cassette within the distal region of the CYT, which is then triggered following IpLITR 1.1b engagement by extracellular targets (Figure 2). In co-immunoprecipitation studies we have also recently observed that following tyrosine phosphorylation, IpLITR 1.1b associates with the non-catalytic region of tyrosine kinase adaptor protein 1 (Nck) [103], WASp family verprolin-homologous protein-2 (WAVE2) [104], and Syk (unpublished observations; Zwozdesky and Stafford). These novel data add to the growing repertoire of molecules that bind to IpLITR 1.1b and, importantly, link the CYT region of IpLITR 1.1b to endogenously available intracellular proteins that regulate actin-dependent phagocytosis.

Receptor-specific recruitment of Nck, WAVE2, and Syk to IpLITR 1.1b helps to clarify how actin-driven membrane remodeling may be controlled by a putative inhibitory receptor. For example, as described for the human phagocytic receptor CEACAM3 [103], WAVE2 may indirectly associate with IpLITR 1.1b using the adaptor Nck that, based on detailed Src homology 2 (SH2)-domain binding studies [105], could interact with the consensus sequence H-I-Y-D-E-V located around Y^433^ in the proximal region of the IpLITR 1.1b CYT [64]. Following or during the recruitment of intracellular effectors responsible for the destabilization of the actin cytoskeleton to the proximal CYT region, Syk may bind the distal CYT region of IpLITR 1.1b by interacting with phosphorylated tyrosines, Y^477^ and Y^499^, that are positioned in two tandem ITIM motifs capable of accommodating both of the SH2 domains of Syk. This atypical mode of Syk recruitment to tandem ITIMs has already been demonstrated for platelet endothelial cell adhesion molecule-1 (PECAM-1) [107]. The tandem ITIMs located within the CYT region of PECAM-1 are spaced such that the tyrosines are separated by twenty-two amino acids. Interestingly, this is also the precise separation between Y^477^ and Y^499^ within IpLITR 1.1b and might explain how Syk also interacts with the distal CYT region (Figure 2). Once recruited to IpLITR 1.1b, we predict that Syk would bind and activate an intracellular Rho-GEF (*i.e.*, Vav or RhoA) that could then directly regulate one of the many known Rho GTPases that are responsible for controlling rapid actin-driven membrane protrusions through activation of Arp2/3 and the WAVE2 complex [106]. Based on our current data, the Rho family GTPases involved are unlikely to be either Cdc42 or Rac1/2/3 [57]. Overall, this proposed mechanism provides the framework for exploring a new mode of ITAM-independent phagocytosis and will be investigated further in our lab. It is also worth noting that this revised model for IpLITR 1.1b-mediated phagocytosis does not include the activity of class I PI3Ks, which is in agreement with our pharmacological data. However, as proposed in Figure 1, class I PI3Ks may still participate in other IpLITR 1.1b-dependent functional responses and more work is still required to reconcile the contribution and dynamics of PI3K involvement during IpLITR 1.1b-mediated phagocytosis. In mammals, FcR-stimulated phagocytosis proceeds through distinctive phases that are dependent on the coordinate activities of several key effector molecules [108]. These phases include an early PI3K-independent stimulation of actin polymerization that drives the extension of the phagocytic cup to capture targets, followed by a PI3K-dependent contractile mechanism that subsequently closes phagosomes around targets [108]. The size of the extracellular target directly influences these processes, as the engulfment of larger particles (*i.e.*, >3 µm) requires accessory signaling events to promote the final stages of target internalization [51,101,109,110,111,112,113]. We hypothesize that IpLITR 1.1b activates the early PI3K-independent phases of the phagocytic process over a broad range of temperatures; however, unlike IpLITR 2.6b/IpFcRγ-L, IpLITR 1.1b-expressing cells are not able to effectively stimulate class I PI3K catalytic activity and therefore generally exhibit a stalled phagocytic phenotype. Stalled phagocytosis has been observed in other studies using cells treated with broad-spectrum pharmacological inhibitors of PI3Ks, and this effect was only evident using large beads (*i.e.*, >3 µm) [51,113]. All together, these data provide another possible distinction between ITAM-mediated phagocytosis and the alternative PI3K-independent phagocytic mode utilized by IpLITR 1.1b.

## 5. Conclusions

In this review, we have described the ongoing characterization of IpLITRs; a highly polymorphic family of teleost immunoregulatory proteins displaying both structural and phylogenetic relationships with repertoires of immune proteins found throughout mammals. Stimulatory and inhibitory IpLITR-types are co-expressed by a variety of myeloid and lymphoid cell-types in catfish and, as reviewed here, they can also regulate cell-mediated cytotoxicity, cytokine secretion, degranulation, and phagocytosis when expressed in representative mammalian immune cell-lines. Although they clearly display potent immunoregulatory potentials, endogenous ligands for IpLITRs are currently unknown and their overall contributions to cellular immunity in teleosts have not yet been established. As it stands, it is possible that members of the IpLITR family may function as teleost FcR-like proteins, regulators of NK cell responses (*i.e.*, NKRs), and as adhesion proteins or pathogen recognition molecules akin to members of the human CEACAM family. However, establishing these functional relationships will depend on further experiments designed to identify IpLITR ligands and explore the precise *in vivo* functions of individual receptors using fish immune cells. Although our work to date has relied on heterologous expression of IpLITRs in mammalian cells, this strategy has allowed us to demonstrate important conserved aspects regarding IpLITR-mediated stimulatory and inhibitory immunoregulatory signaling events and revealed some unanticipated aspects regarding IpLITR signaling versatility. In particular, it is now clear that the signaling mechanisms responsible for IpLITR-mediated immune regulation are both cell- and context-specific. The unique activity of IpLITRs in different mammalian immune cell systems also reinforces the role of protein interaction domains during the evolution of immunoregulatory receptor-based signaling networks. The inherent modularity of important signaling motifs within the transduction machinery allows for complex and novel regulatory behaviors to arise from relatively simple genetic events such as recombination, deletion, or insertion [114]. As a result, classifications of stimulatory or inhibitory receptors must attempt to match functional outcomes to the presence of canonical immunoregulatory domains such as ITIMs or ITAMs. Alternatively, biochemical studies must be aimed at mapping the relative importance of unique domain organizations within receptor variants in order to better understand receptor-specific contributions to the spatiotemporal activation of transduction molecules within different immune cell types. Studies using these methodologies, including those described here using IpLITRs, already suggest that the heterogeneity observed within the intramolecular interactions between unique immunoregulatory receptors and the diverse complement of intracellular effectors likely facilitates intricate tuning of responses through selective signaling dynamics. The presence of diverse multi-receptor systems throughout immune cell lineages further compound the apparent complexity required for the integrated control of innate immunity. At present, we are only beginning to understand the significance of functional plasticity within individual immunoregulatory receptors, including IpLITRs; however, specific details regarding the intracellular mechanisms responsible for the signaling versatility observed are only now emerging. Herein, we propose simple context-specific models for IpLITR-mediated immune cell regulation that require a minimal complement of intracellular signaling components. Importantly, these suggested signaling mediators and many others that may participate in the phagocytic process are highly conserved among vertebrates and in particular for our studies they are very similar between mammals and fish (Table 1). Therefore, the insights gained from our heterologous studies demonstrate thtat IpLITR-mediated responses in mammalian cells feature the same signaling components that are likely present in fish immune cells (Table 1). Further elucidation of these mechanisms will hopefully reveal insights into how immunoregulatory receptor plasticity has evolved and perhaps continue to uncover novel roles for canonical ITIM- and ITAM-encoding receptors. These foundational studies of IpLITR-mediated signaling events have set the stage for future studies targeted at understanding how the dynamic control of intracellular events controlled by immunoregulatory receptors contribute to the conserved activities of innate immune cells across vertebrates.

## Figures and Tables

**Table 1:** Protein basic local alignment search tool (BLASTp) searches for vertebrate immunoregulatory receptor-like sequences and intracellular signaling proteins in the Channel catfish (*Ictalurus punctatus*).

Human FcRLs	Top Matching Catfish Sequences	E value	Score	ID	Pos.	Coverage
**FcRL6** (XP_005245185)	FcRI (NP_001187150)	4e-09	55.8	26%	37%	89%
**FcRL5** (AAK31327)	**LITR TS32.15 L2.1a** (ABI23564)	1e-19	165	26%	40%	90%
**FcRL4** (NP_112572)	**LITR TS32.15 L2.2c** (ABI23567)	3e-11	63.2	25%	38%	89%
**FcRL3** (NP_443171)	**LITR TS32.15 L1.1a** (ABI16036)	1e-18	88.2	25%	39%	90%
**FcRL2** (NP_110391)	**LITR TS32.17 L2.1a** (ABI23578)	6e-06	46.2	24%	39%	79%
**FcRL1 **(NP_443170)	Hepacam 2 (NP_00118775)	2e-04	40.8	23%	46%	38%
**Human FcRs**	**Top Matching Catfish Sequences**	**E value**	**Score**	**ID**	**Pos.**	**Coverage**
**FcyRI** (NP_000557)	FcRI (NP_001187150)	2e-17	79.0	26%	40%	96%
	**LITR TS32.15 L1.1a** (ABI16036)	2e-11	62.4	26%	40%	50%
**FcεRIα** (XP_003821022)	**LITR TS32.17 L1.1a** (ABI16049)	2e-07	48.5	29%	43%	96%
**FcγRIIA** (NP_001129691)	*−no matches−*	−	−	−	−	−
**FcγRIIIA** (NP_001121068)	**LITR TS32.15 L2.1a** (ABI23564)	1e-07	49.7	25%	44%	77%
**FcγRIIC** (NP_963857)	*−no matches−*	−	−	−	−	−
**FcγRIIIB** (NP_001231682)	**LITR TS32.15 L2.1a** (ABI23564)	1e-05	44.3	25%	44%	66%
**FcγRIIB** (NP_001002274)	*−no matches−*	−	−	−	−	−
**FcµR** (AAP36498)	*−no matches−*	−	−	−	−	−
**PIGR** (NP_002635)	*−no matches−*	−	−	−	−	−
**FcαµR** (NP_114418)	*−no matches−*	−	−	−	−	−
**Human LRC Proteins**	**Top Matching Catfish Sequences**	**E value**	**Score**	**ID**	**Pos.**	**Coverage**
**Fcα**R (NP_001991)	*−no matches−*	−	−	−	−	−
**NKp46** (O76036)	*−no matches−*	−	−	−	−	−
**LAIR1** (NP_001275954)	*−no matches−*	−	−	−	−	−
**LILRB1** (AAC51179)	**LITR1** (AAW82352)	6e-10	98.2	25%	39%	66%
**LILRB2** (XP_011546934)	**LITR TS32.15 L2.2c** (ABI23567)	9e-13	68.6	23%	38%	85%
**LILRB3** (O75022)	**LITR1** (AAW82352)	4e-07	50.4	23%	39%	73%
**LILRB4** (NP_001265355)	*−no matches−*	−	−	−	−	−
**LILRB5** (NP_006831)	**LITR1** (AAW82352)	2e-10	60.8	24%	38%	66%
**LILRA1** (NP_006854)	**LITR TS32.15 L2.2c** (ABI23567)	2e-10	61.2	24%	38%	89%
**LILRA2** (XP_011545392)	**LITR1** (AAW82352)	2e-13	70.1	24%	38%	66%
**LILRA3** (Q8N6C8)	**LITR TS32.17 L1.2b** (ABII6052)	4e-09	55.5	24%	40%	53%
**LILRA4** (P59901)	**LITR1** (AAW82352)	2e-08	54.7	26%	40%	65%
**LILRA5** (A6NI73)	*−no matches−*	−	−	−	−	−
**LILRA6** (AGZ61988)	**LITR1** (AAW82352)	1e-09	51.2	23%	38%	68%
**KIR3DL1** (ADM64608)	**LITR3** (NP_001187136)	7e-08	52.4	27%	41%	65%
**KIR3DS1** (ABX88987)	**LITR3** (NP_001187136)	8e-07	48.9	27%	41%	65%
**KIR2DL1** (AAC50335)	*−no matches−*	−	−	−	−	−
**KIR2DS1** (XP_011546300)	*−no matches−*	−	−	−	−	−
**KIR2DL4** (ABW73959)	*−no matches−*	−	−	−	−	−
**Human Siglecs**	**Top Matching Catfish Sequences**	**E value**	**Score**	**ID**	**Pos.**	**Coverage**
**Siglec-1** (Q9BZZ2)	**LITR3** (NP_001187136)	2e-16	137	25%	39%	51%
**Siglec-2** (NP_001762)	**LITR TS32.15 L1.1a** (ABI16036)	7e-18	170	26%	40%	74%
**Siglec-3** (P20138)	*−no matches−*	−	−	−	−	−
**Siglec-4** (NP_002352)	*−no matches−*	−	−	−	−	−
**Siglec-15** (Q6ZMC9)	*−no matches−*	−	−	−	−	−
**Human CEACAMs**	**Top Matching Catfish Sequences**	**E value**	**Score**	**ID**	**Pos.**	**Coverage**
**Ceacam-1** (NP_001703)	Hepacam 2 (NP_00118775)	4e-14	144	31%	44%	64%
	**LITR3** (NP_001187136)	1e-09	58.5	24%	43%	65%
**Ceacam-3** (NP_291021)	Hepacam 2 (NP_00118775)	9e-11	59.3	31%	50%	56%
**Ceacam-4** (NP_001808)	*−no matches−*	−	−	−	−	−
**Ceacam-5** (NP_001295327)	**LITR3** (NP_001187136)	1e-22	166	25%	41%	75%
**Ceacam-6** (P40199)	Hepacam 2 (NP_00118775)	4e-14	71.2	31%	47%	48%
	**LITR TS32.17 L2.2b** (ABI23581)	3e-09	56.2	27%	41%	56%
**Ceacam-7** (Q14002)	Hepacam 2 (NP_00118775)	2e-07	49.7	36%	52%	32%
**Ceacam-8** (NP_001807)	Hepacam 2 (NP_00118775)	6e-10	58.5	27%	42%	51%
	**LITR TS32.15 L2.3b** (ABI23569)	2e-08	53.1	28%	42%	56%
**Mouse PIRs**	**Top Matching Catfish Sequences**	**E value**	**Score**	**ID**	**Pos.**	**Coverage**
**PIRA** (NP_035217)	**LITR TS32.15 L1.1a** (ABI16036)	1e-12	61.6	24%	39%	88%
**PIRB** (AAC53219)	**LITR TS32.15 L1.1a** (ABI16036)	1e-11	105	24%	39%	92%
**Chicken Receptors**	**Top Matching Catfish Sequences**	**E value**	**Score**	**ID**	**Pos.**	**Coverage**
**FcRL1-like** (XP_015135539)	**LITR TS32.17 L2.1a** (ABI23578)	2e-06	46.6	26%	43%	73%
**FcRL2-like** (NP_001090998)	IpFcRI (NP_001187150)	4e-21	91.3	24%	43%	65%
	**LITR3** (NP_001187136)	3e-11	63.2	25%	41%	69%
**CHIR-A** (AAG37067)	**LITR TS32.17 L2.1a** (ABI23578)	5e-09	53.9	28%	45%	75%
**CHIR-B** (AAG37068)	**LITR1** (AAW82352)	6e-10	101	27%	42%	83%
**CHIR-AB1** (NP_001139613)	**LITR TS32.15 L2.2c** (ABI23567)	3e-07	79.7	30%	46%	78%
**Xenopus Receptors**	**Top Matching Catfish Sequences**	**E value**	**Score**	**ID**	**Pos.**	**Coverage**
**FcγRI-L** (XP_012809801)	FcRI (NP_001187150)	1e-18	84.0	34%	45%	63%
	**LITR TS32.15 L2.2c** (ABI23567)	1e-12	67.4	28%	45%	62%
**FcR3-L** (XP_012824905)	FcRI (NP_001187150)	6e-17	80.1	22%	46%	43%
	**LITR TS32.15 L2.3c** (ABI23570)	2e-15	76.6	25%	43%	61%
**FcR4-L** (XP_012809008)	FcRI (NP_001187150)	4e-18	83.2	21%	44%	58%
	**LITR TS32.15 L2.2c** (ABI23567)	6e-15	75.5	23%	40%	92%
**FcR5-L** (XP_012825305)	**LITR TS32.17 L2.1a** (ABI23578)	8e-19	89.7	25%	44%	41%
**ILR-1** (XP_002938564)	**LITR TS32.17 L2.2b** (ABI23581)	2e-12	102	28%	44%	67%
**ILR-2** (NP_001121201)	**LITR TS32.17 L2.1a** (ABI23578)	3e-12	65.1	20%	45%	69%
**Human Signaling Proteins**	**Top Matching Catfish Sequences**	**E value**	**Score**	**ID**	**Pos.**	**Coverage**
**c-Src** (P12931)	Lymphocyte PTP (AJW77401)	0.0	560	59%	77%	82%
**SYK** (P43405)	SYK (NP_998008) *	0.0	863	67%	77%	98%
**PI3K p85α** (NP_852664)	PI3K p85α (AHH41763)	0.0	864	62%	76%	99%
**Csk** (NP_004374)	Csk-like (AJW77401)	3e-105	318	40%	58%	95%
**GRB2** (CAG467401)	GRB2 (NP_001187313)	3e-151	421	91%	95%	100%
**Gab2** (BAA76737)	Gab2 (XP_692935)	0.0	793	63%	75%	99%
**Nck1** (NP_001278928)	Nck1 (NP_001278928) *	0.0	615	77%	87%	100%
**SHP-1** (AAA36610)	SHP-1-like (XP_009290704) *	0.0	783	66%	78%	95%
**SHP-2** (BAA02740)	SHP-2-like (CBX19678) *	0.0	1076	91%	94%	100%
**SHIP-1** (AAB49680)	SHIP-1 (AJK26904)	0.0	1164	56%	67%	99%
**SH2D1A** (NP_001108409)	SH2D1A (NP_001187495)	2e-49	155	64%	76%	89%
**Vav3** (EAW51251)	Vav3 (XP_009296581) *	0.0	1173	68%	80%	100%
**Rac1** (NP_008839)	Rac1 (AD027935)	4e-136	381	94%	98%	100%
**Cdc42** (AAM21109)	Cdc42 (NP_001188177)	2e-134	377	96%	97%	100%
**RhoA** (P61586)	RhoA-like (NP_001187623)	2e-138	387	95%	98%	100%
**Wave2** (P61586)	Wave3 (NP_001074059) *	1e-104	322	44%	53%	100%
	Wave2 (NP_957375) *	2e-104	313	76%	85%	38%
**WASp** (NP_000368)	WASp (NP_956232) *	1e-106	327	54%	69%	59%
**N-WASp** (BAA20128)	N-WASp (NP_001076475) *	3e-145	428	75%	85%	53%

**(A**) The amino acid sequences listed in the left column were used as queries to search the non-redundant protein sequence database for catfishes (taxid:7995) by blastp at http://blast.ncbi.nlm.nih.gov/Blast; (**B**) Other than the human signaling protein queries, all searches we performed using the predicted extracellular regions of the receptor sequences (*i.e.*, predicted TM segments and CYT regions were excluded from the searches); (**C**) For each search result the Expect (E) value reported provides the overall significance of the match with lower values closer to zero being considered more significant. The score indicates quality of the alignment with a higher score associated with a better alignment. This value is calculated using a formula that considers alignment of similar or identical residues, as wells as gaps in the alignment; (**D**) The top matching Channel catfish sequences are listed in the second column from the left and those marked with an * represent queries that did not retrieve a match using catfishes (taxid:7995) but did retrieve a match using Danio (taxid:7954); (**E**) Only sequences with scores >40.0 are reported.

**Table 2:** Protein BLAST searches for human immunoregulatory receptor-like sequences in teleost fishes.

Human Receptors	Teleost Matches	E value	Score	ID	Pos.	Coverage
**FcRL5** (AAK31327)	Zebrafish **CD22** (XP_009293714)	3e-46	184	27%	43%	94%
	Salmon **Sialoadhesin-like** (XP_009293614)	3e-46	184	26%	45%	97%
	Herring **Sialoadhesin-like** (XP_012675080)	8e-33	316	30%	45%	96%
	Cichlid** CD22-like** (XP_014269289)	1e-32	276	28%	47%	76%
	Herring **FcRL5** (XP_012678524)	4e-31	261	25%	40%	94%
**FcγRI** (NP_000557)	Asian arowana **FcRL5** (KPP56756)	6e-21	98.2	29%	44%	91%
	Trout **unamed protien** (CDQ78931)	2e-18	88.6	27%	44%	81%
	Salmon **FcγRI-like** (ACN10126)	4e-18	88.2	28%	42%	81%
	Catfish **FcRI** (NP_001187150)	3e-15	79.0	26%	40%	96%
	Catfish **TS32.15 L1.1a** (ABI16036)	4e-09	62.4	29%	44%	50%
**FcγRIIA** (NP_001129691) *	Herring **FcRL5** (XP_012678524)	7e-10	208	31%	41%	79%
	Yellow Croaker **FcγRII-like** (XP_010752023)	9e-10	60.5	25%	45%	78%
	Tilapia **FcγRIB-like** (XP_013119926)	2e-09	59.7	37%	54%	41%
	Mexican tetra **CD22** (XP_015459483)	3e-09	60.8	31%	47%	70%
	Herring **FcRL3** (XP_012675232)	2e-08	58.9	27%	50%	70%
**FcγRIIB** (NP_001002274) *	Yellow Croaker **FcγRII-like** (XP_010752023)	1e-11	65.9	28%	48%	67%
	Killifish **unknown protein** (XP_013889184)	2e-10	65.1	27%	43%	82%
	Mexican tetra **CD22** (XP_015459483)	4e-10	63.9	31%	46%	61%
	Herring **FcRL3** (XP_012675232)	2e-09	62.0	28%	51%	70%
	Cichlid **FcγRII-like** (XP_006808904)	3e-08	58.2	28%	42%	76%
**FcµR** (AAP36498) *	Damselfish **unknown protein** (XP_008286766)	4e-06	52.8	34%	47%	42%
	Salmon **CMRF35-like** (XP_014058627)	8e-06	51.2	34%	47%	42%
	Killifish **PIGR-like** (012722396)	2e-05	50.1	29%	41%	40%
**PIGR** (NP_002635) *	Tilapia **PIGR-like** (XP_013123119)	4e-25	115	27%	44%	67%
	Yellow **Croaker PIGR** (KKF27361)	1e-23	111	25%	39%	81%
	Medaka **PIGR** (XP_011484914)	3e-23	262	28%	44%	72%
	Carp **PIGR** (ADB97624)	1e-18	92.8	31%	47%	73%
	Zebrafish **PIGR** (NP_001289179)	7e-18	90.5	30%	47%	68%
**FcαR** (NP_001991) *	Cichlid **unknown protein** (XP_014265321)	7e-05	48.5	29%	46%	51%
**NKp46** (O76036) *	Atlantic molly **CD276-like** (XP_014823336)	4e-05	49.7	24%	37%	65%
	Sailfin molly **IgSF 1-like Rc** (XP_014882122)	4e-04	46.6	24%	38%	69%
**LAIR1** (NP_001275954) *	*−no matches−*	−	−	−	−	−
**LILRB1** (AAC51179)	Pike **DSCAM-like** (XP_12987993)	1e-15	84.3	28%	41%	85%
	Pike **FcRL5** (XP_010867787)	6e-14	79.7	26%	43%	82%
	Salmon **IgSF 1-like Rc** (XP_01401166)	2e-11	71.6	27%	41%	83%
	Pike **LILRA4-like** (XP_012988338)	4e-10	64.3	28%	44%	42%
	Cafish **LITR1** (AAW82352)	2e-09	65.1	26%	40%	86%
**LILRB4** (NP_001265355) *	Cichlid **IgSF 1-like Rc** (XP_014267965)	2e-11	68.9	27%	47%	78%
	Amazon molly **PEACAM-like** (XP_007571377)	4e-07	56.2	28%	42%	77%
	Atlantic molly **IgSF 1-like Rc** (XP_014827576)	1e-05	51.6	29%	45%	78%
	Yellow Croaker **LILRA2-like** (KKF09113)	2e-05	51.2	31%	45%	64%
**LILRA2** (XP_011545392)	Trout **unamed protien** (CDQ78931)	1e-12	75.9	27%	42%	90%
	Pike **DSCAM-like** (XP_12987993)	5e-12	73.6	27%	42%	82%
	Catfish **LITR1** (AAW82352)	4e-11	70.1	24%	38%	66%
	Mexican tetra **FcRL5** (XP_015464057)	8e-11	69.7	28%	41%	72%
	Pike **FcRL5** (XP_010867787)	7e-10	66.7	27%	42%	77%
**LILRA5** (A6NI73) *	Pllatyfish **IgSF 1-like Rc** (XP_005816340)	4e-06	53.1	28%	40%	72%
	Cichlid **KIR2DL4-like** (XP_005951820)	4e-05	48.9	35%	48%	40%
	Cichlid **FcγRI-like** (XP_014267764)	6e-05	48.5	34%	49%	40%
	Cichlid **IgSF 1-like Rc** (XP_014267965)	3e-04	47.0	32%	45%	44%
**KIR3DL1** (ADM64608)	Asian arowana **FcRL5** (KPP56756)	1e-11	70.5	25%	42%	63%
	Salmon **KIR3DL1-like** (XP_014042396)	2e-11	69.7	30%	43%	85%
	Cichlid **IgSF 1-like Rc** (XP_014267965)	9e-08	70.5	25%	43%	85%
	Salmon **KIR3DS1-like** (XP_014043811)	2e-07	55.5	31%	44%	56%
	Catfish **LITR3** (NP_001187136)	1e-05	52.0	27%	41%	65%
**KIR2DL1** (AAC50335) *	Salmon **KIR3DS1-like** (XP_014043811)	1e-05	49.7	28%	40%	78%
	Salmon **KIR3DL1-lik**e (XP_014042396)	4e-05	49.3	28%	40%	78%
	Salmon **LILRA4-like** (XP_014037968)	8e-05	48.5	29%	41%	75%
	Pike **OSCAR-like** (XP_012988534)	1e-04	47.8	29%	44%	81%
	Pike **DSCAM-like** (XP_12987993)	1e-04	48.1	26%	43%	81%
**Siglec-2** (NP_001762)	Zebrafish **CD22** (XP_009293714)	8e-71	738	30%	45%	99%
	Cichlid **CD22-like** (XP_005755784)	4e-64	221	33%	52%	67%
	Tilapia **CD22-like** (XP_013132285)	6e-61	218	30%	46%	85%
	Trout **unamed protien** (CDQ84455)	2e-58	212	31%	47%	86%
**Siglec-4** (NP_002352) *	Salmon **Siglec-4 (MAG)** (XP_014021122)	4e-154	459	44%	64%	96%
	Pike **Siglec-4 (MAG)** (XP_014021122)	7e-152	454	43%	63%	96%
	Black cod **Siglec-4 (MAG)** (XP_010788688)	4e-151	452	44%	63%	96%
	Tiger puffer **Siglec-4 (MAG)** (NP_001027876)	2e-149	449	43%	63%	96%
**Ceacam-3** (NP_291021)	Herring **Hemicentin-1-like** (XP_012676907)	1e-18	90.5	34%	52%	65%
	Cichlid **CEACAM 5-like** (XP_014266060)	4e-16	82.8	33%	53%	82%
	Salmon **CEACAM 1-like** (XP_014038441)	1e-15	79.3	33%	52%	55%
	Medaka **CEACAM 1-like** (XP_011483887)	5e-15	77.8	28%	42%	94%
**Ceacam-4** (NP_001808) *	*−no matches−*	−	−	−	−	−
**Ceacam-5** (NP_001295327)	Tongue sole **CEACAM 5-like** (XP_008322222)	4e-80	277	30%	50%	85%
	Yellow Croaker **CEACAM 5** (KKF27703)	4e-75	918	32%	53%	98%
	Pike **CEACAM 5** (XP_012994902)	4e-69	570	32%	48%	86%
	Cichlid **Hemicentin-1-like** (XP_004550953)	1e-65	892	30%	47%	83%
	Salmon **Hemicentin-1-like** (XP_014056825)	3e-65	1041	30%	46%	86%

**(A**) The human receptor amino acid sequences listed in the left column were used as queries to search the non-redundant protein sequence database for teleost fishes (taxid:32443) by blastp at http://blast.ncbi.nlm.nih.gov/Blast; (**B**) Human receptor sequences marked with an * indicate those that did not retrieve matches by blastp searches using catfishes (taxid:7995); see Table 1; (**C**) All searches we performed using the predicted extracellular regions of the receptor sequences (*i.e*., predicted TM segments and CYT regions were excluded from the searches); (**D**) For each search result the Expect (E) value reported provides the overall significance of the match with lower values closer to zero being considered more significant. The score indicates quality of the alignment with a higher score associated with a better alignment. This value is calculated using a formula that considers alignment of similar or identical residues, as wells as gaps in the alignment; **(E**) Representative top-matching teleost protein sequences are listed in the second column from the left; (**F**) Only sequences with scores >40.0 are reported.

## Abbreviations

Abs	antibodies
Akt	protein kinase B
CEACAM	carcinoembryonic antigen-related cell adhesion molecule
Csk	C-terminal Src kinase
CYT	cytoplasmic tail
EAT-2	Ewing’s sarcoma (EWS)-activated transcript 2
ERK	extracellular signal-regulated kinase
FcR	Fc receptor
FcRL	FcR-like protein
FcRg	FcR common gamma chain
Gab	Grb2-associated binder
GEF	guanine nucleotide exchange factors
Grb2	growth factor receptor-bound 2
HA	hemagglutinin
IgSF	immunoglobulin superfamily
ITAM	immunoreceptor tyrosine-based activation motif
ITIM	immunoreceptor tyrosine-based inhibition motif
ITSM	Immunoreceptor tyrosine-based switch motif
IL	interleukin
JNK	c-Jun N-terminal kinase
KIR	killer Ig-like receptor
LILR	leukocyte Ig-like receptor
LITR	leukocyte immune-type receptor
LRC	leukocyte receptor complex
mAb	monoclonal antibody
ERK	mitogen activated protein kinase
NITR	novel immune-type receptor
NK	natural killer
NKRs	NK receptors
PH	pleckstrin homology
PI3K	phosphatidylinositol 3-kinase
PI(3,4,5)P3	phosphatidylinositol 3,4,5-trisphosphate
PTK	protein tyrosine kinases
PKC	protein kinase C
RBL	rat basophilic leukemia
SFK	Src family kinase
SHP	SH2 domain-containing cytoplasmic phosphatases
SH2D1A	SH2 domain protein 1A
SHIP	SH2-domain containing inositol 5-phosphatase
SoS	Son of Sevenless
Syk	spleen tyrosine kinase
TM	transmembrane
TNF-α	tumor necrosis factor-alpha
WAVE2	WASp family verprolin-homologous protein-2
